# Association between environmental mold exposure and mild cognitive impairment in older adults: a case–control study

**DOI:** 10.3389/fpubh.2026.1877177

**Published:** 2026-07-13

**Authors:** Raquel Rivera-Carvajal, Diana Carolina Tiga-Loza, D. Jimena Roncancio, Laura Andrea Rodriguez-Villamizar, Gisela Ríos-Gajardo, Alima Valentina Ardila-Alvarez, Nicole Karim Suárez-Acosta, Beatriz Elena Guerra-Sierra

**Affiliations:** 1 Facultad de Ciencias Médicas y de la Salud, Doctorado en Enfermedades Infecciosas, Instituto de Investigación MASIRA, Universidad de Santander, Bucaramanga, Colombia; 2 Grupo Agua, Salud y Ambiente, Facultad de Ingeniería, Universidad El Bosque, Bogotá, Colombia; 3 Universidad Industrial de Santander, Bucaramanga, Colombia; 4 Universidad de Concepción, Concepción, Chile; 5 Universidad Autónoma de Bucaramanga, Bucaramanga, Colombia; 6 Facultad de Ciencias Naturales, Universidad de Santander, Bucaramanga, Colombia

**Keywords:** indoor dampness, environmental molds, indoor air quality, sick building syndrome, older adults, environmental assessment, dampness and mold assessment tool, cognitive decline

## Abstract

**Introduction:**

Cognitive disorders have been associated with exposure to environmental molds in case series and animal experimental models. Older adults spend a substantial proportion of their time indoors, where they may be exposed to household mold contamination.

**Objective:**

To determine the association between indoor environmental mold exposure and amnestic mild cognitive impairment (aMCI) among older adults in Bucaramanga during 2024–2025.

**Methods:**

A case–control study (1:1 ratio) was conducted, including 60 cases and 60 controls, matched by sex, age, and neighborhood of residence. Cases were selected from the INTERCOG study and controls from the general population. Data collection included participant and housing characterization, the Dampness and Mold Assessment Tool for General Buildings (DMAT), and an assessment of Sick Building Syndrome (SBS) related symptoms. Air culture samples were collected from bedrooms, living rooms, and kitchens. Indoor CO_2_, relative humidity, and temperature, mold-specific serum IgE levels were measured. Multivariable analyses were performed using conditional logistic regression.

**Results:**

Most participants were female, with a median age of 69 years. aMCI was significantly associated with increased odds of SBS-related nasal irritation (OR = 5.84; 95%CI: 1.20–28.37). The presence of *Fusarium* spp. in kitchen samples (OR = 3.86; 95%CI: 0.85–17.30) and history of diabetes mellitus (DM) showed a positive but non-significant association with aMCI (OR = 7.68; 95%CI: 0.95–61.16). Higher indoor temperature and greater frequency of household cleaning exhibited a protective trend against aMCI.

**Conclusion:**

Nasal irritation experienced indoor and relieved upon leaving the home was significantly associated with aMCI. Positive but non-significant associations were observed for the presence of *Fusarium* spp. and a history of DM. Higher indoor temperature and more frequent household cleaning showed inverse, non-significant associations with aMCI. These findings highlight the need for further research to elucidate the role of residential environmental exposures with aMCI.

## 1 Introduction

Mild cognitive impairment (MCI) involves deficits in cognitive functions such as immediate, remote, episodic, and working memory, as well as alterations in attention, language, orientation, psychomotor functions, and sleep disturbances. It is also considered a transitional state preceding more severe conditions such as Alzheimer's disease (AD) (1). Globally, the prevalence of MCI is estimated to range from 3 to 19% among individuals aged ≥65 years (2). In Latin America, reported prevalence ranges from 6.8 to 25.2% among adults aged ≥60 years (3). The world population is aging, and the MCI prevalence increases with age (3). Global estimates indicate that the population ≥65 years will reach approximately 1.4 billion by 2030 and 2.1 billion by 2050 (4). In addition, the development of MCI is associated with substantial direct and indirect costs (5, 6).

MCI is associated with a reduced quality of life, affecting physical, psychological, and social dimensions, including instrumental activities of daily living. Over time, it leads to progressive declines in autonomy, the ability to influence one's future, intimacy, and the capacity to experience love and affection (7). These changes may contribute to increased levels of stress, depression, and emotional distress at both the individual and family levels (8). Furthermore, as cognitive decline compromises patient autonomy, family members and caregivers must be prepared to assume increasing responsibilities, including the management of treatments and therapies, as well as support with feeding, dressing, hygiene, and overall daily care (9).

Identifying the underlying etiology of MCI is crucial, as it has been estimated that between 30 and 50% of cases may be reversible (1). MCI is classified according to memory involvement into amnestic and non-amnestic subtypes, which can be further subdivided into single-domain and multiple-domain forms, depending on the cognitive domains affected, including memory, executive function, language, and attention (10). The non-amnestic subtype has been associated with distinct neuropathological processes, including frontotemporal degeneration, vascular changes, and Lewy body disease (11). Therefore, identifying associated factors may provide opportunities to implement targeted interventions aimed at preventing progression to dementia.

**Figure 1:**
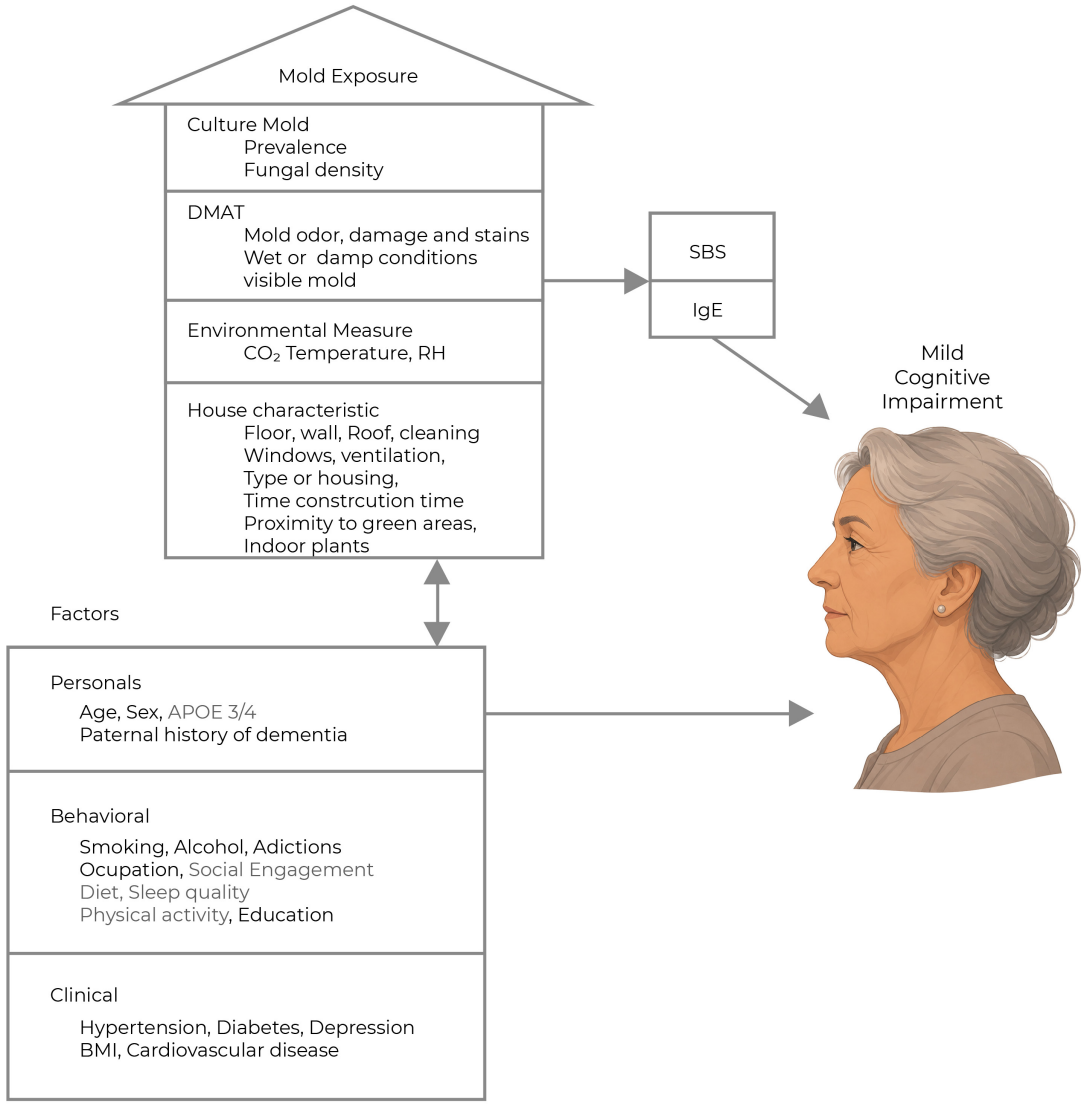
Factors evaluated for their association with amnesic mild cognitive impairment in the study. DMAT, Dampness and Mold Assessment Tool; BMI, body mass index; APOE, apolipoprotein E; CO_2_, carbon dioxide; RH, relative humidity; SBS, sick building syndrome; IgE, immunoglobulin E. Dark gray indicates measured variables, while light gray indicates variables not measured.

Factors associated with the development of MCI are multifactorial (see Figure 1) and include genetic, sociodemographic, lifestyle, and clinical determinants. Genetic factors include the presence of the *APOE*ε4 allele (12). Sociodemographic factors encompass advanced age, lower educational attainment, marital status (separated, widowed, or single), and female sex. Lifestyle and clinical factors include body mass index (overweight/obesity), depressive symptoms, insomnia, frailty, sarcopenia, alcohol consumption, smoking, oral health problems (periodontitis and gingivitis), hypertension, diabetes, and infections, among others (13, 14).

Infectious processes have been proposed as potential triggers of cognitive impairment and even AD. This hypothesis dates back to the early characterization of AD by Alois Alzheimer more than a century ago (15). Although subsequent etiological research primarily focused on vascular and genetic factors, there has been renewed interest in the role of infections in the pathogenesis of AD. In particular, microorganisms including viruses, bacteria, fungi, or their combined interactions have been implicated in the formation of β-amyloid plaques (16–18). Several viral infections have been associated with cognitive alterations, including human immunodeficiency virus (HIV), herpes simplex virus type 1, varicella-zoster virus, Japanese encephalitis virus, West Nile virus, viruses causing aseptic meningitis, influenza virus, and more recently, SARS-CoV-2 (2).

Fungal pathogens have also been linked to cognitive impairment. Recent studies have identified fungal components in the brain tissue of patients with AD, including proteins such as enolase, β-tubulin, and chitin, as well as fungal DNA (19, 20). Notably, species such as Candida albicans and members of the genus Malassezia, commonly found as commensals of the oral cavity and skin, have been detected in these tissues (19). In the context of indoor environmental molds, genera such as *Aspergillus, Penicillium, Fusarium, Claviceps*, and *Alternaria* have been associated with adverse health effects. Exposure to their conidia and mycotoxins through inhalation, ingestion, or dermal contact has been linked to the induction of proinflammatory cytokine release, which may trigger neuropsychological changes, including cognitive impairment (21).

Several studies have explored the potential relationship between mold exposure and disorders of the central nervous system (CNS) (22–24). However, methodological limitations have been noted (25), leading some authors to conclude that the current evidence is insufficient to establish mold exposure as a definitive risk factor. Nevertheless, in recent years, *in vivo* studies have provided emerging evidence of neurological effects associated with mold exposure. Experimental studies in animal models have demonstrated adverse outcomes following exposure to molds such as *Aspergillus* (26) and *Stachybotrys* (27), as well as to mycotoxins including *aflatoxin B1* (28).

Fungi proliferate in buildings with deficiencies in water supply and sewage systems, as well as in environments with inadequate ventilation, contributing to indoor air contamination. Environmental factors such as high ambient humidity, the use of air conditioning systems, poor hygiene, and insufficient natural lighting further promote fungal growth. These conditions have been recognized as contributing factors to sick building syndrome (SBS), a condition defined by the World Health Organization (WHO) in 1982 (29, 30).

Accordingly, the aim of this study was to determine the association between indoor environmental mold exposure and amnestic aMCI among older adults in the Metropolitan Area of Bucaramanga during 2024–2025.

## 2 Materials and methods

### 2.1 Study design

A matched case–control study with a 1:1 ratio was conducted among older adults residing in the Metropolitan Area of Bucaramanga.

### 2.2 Definition of cases and controls

Cases were defined as individuals aged ≥60 years with a diagnosis of amnestic mild cognitive impairment (aMCI), either single-domain or multiple-domain, confirmed through clinical evaluation by professionals in neuropsychology, psychiatry, and neurology, supported by standardized neuropsychological tests. Severe depression was excluded. Cases were selected from participants at baseline of the INTERCOG study (31), which aims to study the efficacy of a multicomponent intervention on cognitive function for the caregiver-patient dyad (Clinical Trials: NCT06408103). The INTERCOG study participants were required to reside in households within the Metropolitan Area of Bucaramanga, have lived at the same address for at least the previous 2 years, and not be institutionalized.

Controls were individuals aged ≥60 years, matched to cases by sex, age (±5 years), and neighborhood of residence [usually with the same socioeconomic stratum (32)]. They were required to live in private households, not be institutionalized, reside in a different household from the case, and have lived at the same address for at least the previous 2 years. Individuals with terminal illnesses (e.g., cancer or cerebrovascular disease) were excluded. Potential controls were identified through referral by cases (convenience sampling) and subsequently underwent cognitive assessment using the Montreal Cognitive Assessment (MoCA) to confirm the absence of cognitive impairment (33).

### 2.3 Study population and sample size

The sample size was estimated at 120 participants, including 60 cases with aMCI and 60 controls without cognitive impairment, with a 1:1 matching ratio by sex, age (±5 years), and neighborhood of residence. Sample size and statistical power were calculated based on previously reported measures of association and mean differences in scale scores between individuals exposed and unexposed to molds, with outcomes related to cognitive impairment. For the present study, a total sample size of 120 participants (60 cases and 60 controls) was determined based on an odds ratio (OR) of 2.94 (95% CI: 2.29–3.79) reported by Zhang et al. (34), assuming a significance level (α) of 0.05 and a statistical power of 0.80. Additional calculations based on other reported outcomes suggested smaller required sample sizes (< 60 participants per group), yielding statistical power estimates above 0.80. However, based on the vocabulary test reported by Kilburn (35), a larger sample size of 118 participants per group would be required; therefore, for the current sample of 60 participants per group, the estimated statistical power for this specific outcome was 0.51.

### 2.4 Instruments

#### 2.4.1 Cognitive function

Cognitive function was assessed by a team of neuropsychology professionals using a battery of tests validated in Colombia for the identification of aMCI (36) (Mini-Mental State Examination, Montreal Cognitive Assessment, Hopkins Verbal Learning Test, Digit Symbol Test, Trail Making Test Parts A and B, Verbal Fluency Test, Stroop Color and Word Test, Rey–Osterrieth Complex Figure Test, and Token Test. See Supplementary Table 1). Participants were also evaluated by a neurologist to rule out organic causes potentially associated with aMCI and by a psychiatrist to exclude psychiatric conditions that could account for cognitive impairment.

#### 2.4.2 Characterization of participants

A participant characterization form was used to collect sociodemographic data (sex, age, educational level, place of origin, place of residence, marital status, occupation, type of work, and socioeconomic status), medical history (myocardial infarction, angina, cancer, diabetes mellitus, hypertension, and cerebrovascular disease), and lifestyle factors, including smoking status and use of biomass fuels for cooking. Physical measurements included weight, height, body mass index (BMI), systolic blood pressure, and diastolic blood pressure.

#### 2.4.3 Housing and environmental characteristics

A structured questionnaire was used to collect housing characteristics, including type of dwelling, tenure status, predominant construction materials of floors, walls, and roofs, total floor area (m^2^), and number of rooms. The questionnaire also captured factors potentially associated with indoor dampness and mold, such as year of housing construction, presence of indoor plants, cleaning frequency (Responses ranged from 1 to 7 days per week and were categorized into three groups according to cleaning frequency: once per week, 2–3 times per week, and ≥4 times per week), pet ownership (dogs and cats), proximity to green areas (e.g., parks), predominant topography, window conditions, perceived lighting, and the functional condition of roofs, walls, and water supply and sewage systems.

The condition of windows, roofs, walls, water supply, and sewage systems was classified as poor (frequent damage), fair (occasional damage or delayed repairs), good (rare damage or prompt repairs), or excellent (no history of damage).

#### 2.4.4 Assessment of dampness and mold exposure

Exposure to indoor dampness and mold was assessed using the Dampness and Mold Assessment Tool (DMAT), which was translated into Spanish, culturally adapted for Latin American and Spanish-speaking settings, and validated by an expert panel as part of the present study protocol. Details of the validation process have been reported elsewhere (37, 38). The DMAT generates a semi-quantitative score based on the presence of mold odor, visible mold, damage, and moisture across multiple housing components and rooms.

The extent of damage was quantified using the following scoring system: 0 = no evidence of damage; 1 = damage smaller than the size of a standard sheet of paper; 2 = damage between the size of a standard sheet of paper and a standard door; and 3 = damage larger than the size of a standard door. Scores were assigned upon identification of damage and stains (including water stains, peeling paint, efflorescence, rust, material deformation, or crumbling), visible mold (spots or discoloration differing from the underlying material, typically gray, brown, or black), and wet or damp conditions (visible signs of moisture, water droplets, leaks, or flooding). Assessments were conducted during home visits by trained evaluators using a standardized instruction manual with detailed operational definitions and illustrative examples (37).

#### 2.4.4 Assessment of sick building syndrome symptoms (SBS)

SBS was assessed using a questionnaire evaluating the presence of symptoms occurring while at home and improving upon leaving the dwelling (39–41). The following symptoms were investigated: general symptoms (fatigue/heaviness in the head, dizziness/nausea, difficulty concentrating, and shortness of breath); skin symptoms (dry or flushed facial skin, itchy scalp, and itchy hands); and mucosal symptoms (eye irritation, nasal irritation, and throat dryness). Response options were categorized as never (0), rarely (>2 weeks), sometimes (1–2 times per week), and often (every week). Symptoms were considered present when participants reported experiencing them often (every week).

#### 2.4.5 Culture of air samples for the enumeration of colony-forming units (CFU)

Air sampling for CFU enumeration was conducted following previously described methodologies (42). Sampling was performed in households using a MAS-100 air sampler (Merck, Darmstadt, Germany), which complies with the criteria established in ISO/CD 14698-1 (43). Air samples were collected at a flow rate of 200 L/min for 2 min using malt extract agar supplemented with chloramphenicol (0.05 g/L). Sampling was conducted at a height of 1 m above the floor using a tripod, in three locations within each household: the living room, kitchen, and the older adult's bedroom, where occupants were assumed to spend most of their time during the day (44, 45). Samples were subsequently transported under cold chain conditions to the Laboratory of Research and Innovation in Agro-environmental Biotechnology (LIIBAAM_UDES) for processing. Plates were incubated at 25 °C and monitored for growth every 24 h. Once colonies were established, CFU counts were recorded using a data collection instrument designed in REDCap. Fungal density was expressed as colony-forming units per cubic meter (CFU/m^3^), calculated using the formula: CFU/m^3^ = number of colonies / (sampled volume in liters / 1,000).

Permits and Regulatory Compliance: This study was conducted under the Framework Permit for the Collection of Wild Specimens of Biological Diversity for Non-Commercial Scientific Research granted to Universidad de Santander (UDES) by the National Environmental Licensing Authority (ANLA), Colombia (Resolution No. 01749 of December 29, 2017; valid until 2027). Fungal isolates recovered from indoor environments, including potentially mycotoxigenic species, were processed, identified, and reported in accordance with the permit requirements and applicable Colombian environmental regulations.

#### 2.4.6 Measurement of mold-specific IgE

Serum samples were collected to determine specific IgE levels against environmental molds, including *Alternaria alternata/Alternaria* tenuis, *Aspergillus fumigatus (*m3), and *Penicillium chrysogenum* (m1). Samples were stored under frozen conditions at the LIIBAAM laboratory (UDES) and subsequently processed by a certified external laboratory (SYNLAB) following standardized protocols for sample preservation and transport. IgE quantification was performed using the fluorescence enzyme immunoassay (FEIA) technique (ImmunoCAP), which measures circulating allergen-specific antibodies. The following allergens were analyzed: rAlt a 1 (3664-m229; *Alternaria alternata/tenuis)*, allergen extract for *Alternaria alternata/tenuis* (64044 [1729]), *Aspergillus fumigatus* (m3; 64019 [1744]), and *Penicillium chrysogenum* (m1; 64356 [1962]; *Penicillium notatum*). A minimum of 0.5 ml of serum was required. Sample stability was maintained for up to 1 week at room temperature (15–25 °C), 2 weeks under refrigeration (2–8 °C), and up to 1 month at −20 °C, provided that samples were centrifuged promptly and aliquoted into polypropylene tubes free of hemolysis. Reference ranges for specific IgE levels (kU/L) were classified as follows: negative (Class 0: < 0.35); positive—Class 1: 0.35–0.70; Class 2: 0.71–3.59; Class 3: 3.6–17.0; Class 4: 17.1–50.0; Class 5: 50.1–100; and Class 6: >100 (46).

#### 2.4.7 Environmental measurements

Environmental parameters, including temperature, relative humidity, and carbon dioxide (CO_2_), were measured using portable devices. Temperature and relative humidity were assessed using a CENTER 316 Humidity Temperature Meter (Center Technology Corp), with a measurement range of −20 °C−60°C for temperature and 0%−100% for relative humidity. CO_2_ concentrations were measured using portable devices (Aranet4 Home and Pro; Aranet Wireless Solutions, Spain), which are battery-powered wireless sensors. CO_2_ levels were used as an indicator of indoor ventilation and air exchange within households. CO_2_ concentrations were interpreted as follows: outdoor levels typically range from 250 to 350 ppm, while indoor concentrations between 350 and 1,000 ppm are considered acceptable. Levels between 1,000 and 2,000 ppm indicate poor indoor air quality, and concentrations exceeding 2,000 ppm are considered indicative of stale air and may pose potential health risks (47, 48). Environmental measurements were analyzed as continuous variables.

### 2.5 Data collection

Home visits were conducted between August 2024 and December 2025. Data collection was carried out using REDCap (49), installed on a Universidad de Santander server. Photographs of areas with locative damage were taken to verify the scores assigned to the extent of damage. Prior to fieldwork, collaborators responsible for conducting the home visits received standardized training on the use of the assessment instrument in its Spanish version and on completion of the data collection forms.

### 2.6 Statistical analysis

In the descriptive analysis, categorical variables were summarized using absolute and relative frequencies, while continuous variables were described using measures of central tendency and dispersion. Normality was assessed using the Shapiro Francia test. Variables with a normal distribution were reported as mean and standard deviation (SD), whereas non-normally distributed variables were presented as median and interquartile range (IQR). In the bivariate analysis, statistical tests were used to compare differences between groups. For categorical variables, Pearson's chi-square test or Fisher's exact test was applied, as appropriate. For continuous variables, Student's *t*-test was used for normally distributed data, and the Mann–Whitney *U*-test for non-normally distributed data.

Multivariable conditional logistic regression models (50) were fitted to identify factors associated with aMCI. Variables with a *p*-value < 0.25 in the crude (bivariate) analysis were considered for inclusion in the multivariable models, alongside variables consistently reported in the literature as relevant predictors. The models inherently accounted for the matching variables (age, sex, and neighborhood of residence) through the study design. Model specification and selection followed the purposeful selection strategy described by Hosmer and Lemeshow (51).

### 2.7 Ethical considerations

The study complied with the ethical principles established in the Declaration of Helsinki and with national regulations governing health research. All participants provided written informed consent before data collection, and the confidentiality and anonymity of the information obtained were ensured. Ethical approval was granted by the Bioethics Committee of the Universidad de Santander, Bucaramanga, Colombia (approval record No. VII-004-BUC, February 8, 2024).

## 3 Results

### 3.1 General characteristics of the study population

Among the 120 participants (60 cases with mild cognitive impairment and 60 controls without cognitive impairment), most were female, with a median age of 69 years (IQR: 63–77). No significant differences were observed in sociodemographic variables between groups (all *p* > 0.05). However, regarding occupation, a higher proportion of retired participants was observed among cases (41.38% [24]) compared with controls (27.59% [16]), whereas being employed or self-employed tended to be more frequent among controls (*p* = 0.057) (see Table 1).

**Table 1:** Sociodemographic characteristics of the participants % (*n*).

Variable	Total (120)	Controls (60)	Cases (60)	*p*-value
Sex	
Female	73.33 (88)	73.33 (44)	73.33 (44)	1.000
Male	26.67 (32)	26.67 (16)	26.67 (16)	
Age, median (IQR)	69 (63, 77)	66 (63, 75)	72 (63, 78)	0.1454
Educational level	
None, primary	30.00 (36)	30.00 (18)	30.00 (18)	0.908
Secondary	31.67 (38)	33.33 (20)	30.00 (18)	
Technical, undergraduate, graduate	38.33 (46)	36.67 (22)	40.00 (24)	
Marital status	
Married/common-law	20.00 (24)	16.67 (10)	23.33 (14)	0.564
Single, separated,	60.00 (72)	60.00 (36)	60.00 (36)	
Widowed	20.00 (24)	23.33 (14)	16.67 (10)	
Occupation	
Housekeeper	47.41 (55)	44.43 (26)	50.00 (29)	0.057
Employed	7.76 (9)	12.07 (7)	3.45 (2)	
Self-employed	10.34 (12)	15.52 (9)	5.17 (3)	
Retired	34.48 (40)	27.59 (16)	41.38 (24)	
Household social strata classification	
Low	19.17 (23)	18.33 (11)	20.00 (12)	0.99
Middle	68.33 (82)	70.00 (42)	66.67 (40)	
High	12.50 (15)	11.67 (7)	13.33 (8)	
Medical history	
Diabetes mellitus	19.17 (23)	15.00 (9)	23.33 (14)	0.246
Hypertension	45.83 (55)	48.33 (29)	43.33 (26)	0.583
COVID-19	32.50 (39)	35.00 (21)	30.00 (18)	0.559
Family history of dementia	24.17 (29)	16.67 (10)	31.67 (19)	0.157
BMI. Mean ± SD	26.33 ± 4.38	27.53 ± 4.41	25.09 ± 4.02	0.0022
Smoking history	31.67 (38)	26.67 (16)	36.67 (22)	0.239

BMI, Body mass index; IQR, interquartile range. p-values were obtained using Pearson's chi-square test, Fisher's exact test, Mann–Whitney U-test, or Student's t-test, as appropriate.

Regarding medical history, a higher frequency of diabetes, family history of Alzheimer's disease, and history of smoking was observed among cases. Mean BMI was higher in the control group compared with the case group.

### 3.2 Perceived symptoms related to SBS

**Figure 2:**
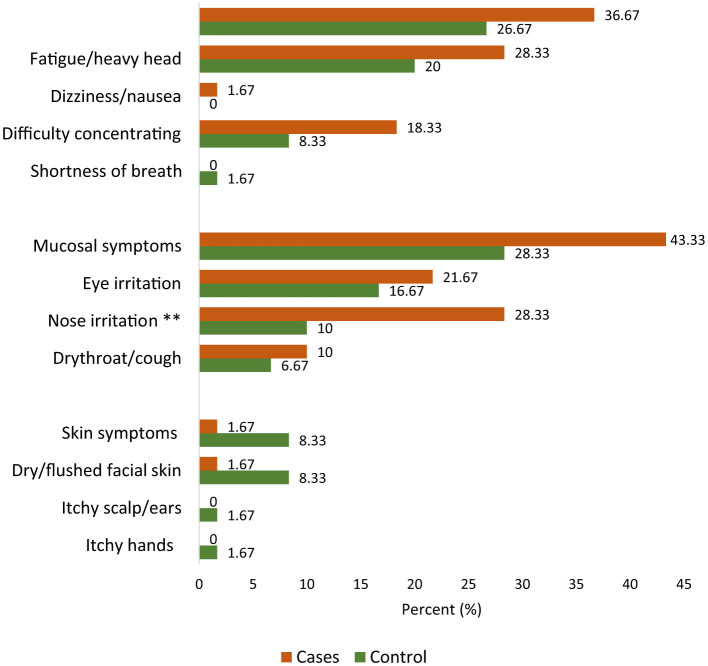
Perceived symptoms related to sick building syndrome. **p-value Pearson's chi-square test: Nose irritation = 0.011, Mucosal symptoms = 0.087.

Mucosal symptoms were the most frequently reported, followed by general symptoms, while skin-related symptoms had the lowest frequency. Among mucosal symptoms, a significant difference was observed for nasal irritation, which was reported by 28.33% (17) of cases and 10.00% (6) of controls (*p* = 0.011) (see Figure 2).

### 3.3 Housing characteristics

Regarding housing characteristics, most dwellings had tile flooring, cement walls, and cement roofing. In terms of housing type, 60.00% (36) of cases lived in apartments compared with 45.00% (27) of controls (*p* = 0.100). Cleaning frequency of once per week was reported by 50.00% (30) of cases and 35.00% (21) of controls (*p* = 0.151). Fungal density differed significantly between groups, with a median of 560 CFU/m^3^ (IQR: 353–837) among cases and 703 CFU/m^3^ (IQR: 505–1,071) among controls (*p* = 0.016). Detailed structural housing conditions, including window, roof, wall, water supply, and sewage system characteristics, are presented in Supplementary Table 2. Comparisons between cases and controls revealed no statistically significant differences. Notably, more than 90% of households in both groups were classified as having good or excellent structural conditions.

Regarding DMAT conditions, higher mean scores were observed among cases than controls for mold odor, damage/stains, and visible mold. However, a statistically significant difference was identified only for wet/damp conditions, with a mean score of 1.21 ± 2.92 among cases and 0.36 ± 0.78 among controls (*p* = 0.0315) (see Table 2).

**Table 2:** Housing characteristics of the participants. %( *n*).

Variable	Total (120)	Controls (60)	Cases (60)	*P*-value
Length of residence in the home (years)	10.5 (4, 20)	12 (5.5, 22.5)	10 (3, 20)	0.1888
Housing construction time, years	30 (15.5; 45)	30.5 (20; 45)	26 (12; 45)	0.3513
Floor material	
Cement	32.50 (39)	36.67 (22)	28.33 (17)	0.398
Tile	66.07 (80)	63.33 (38)	70.00 (42)	
Wall material	
Cement	57.50 (69)	60.00 (36)	55.00 (33)	0.830
Wood	1.67 (2)	1.67 (1)	1.67 (1)	
Brick	38.33 (46)	38.33 (46)	41.67 (25)	
Roof material	
Cement	79.77 (95)	83.33 (50)	75.00 (45)	0.517
Fiber cement (Eternit)	11.67 (14)	11.67 (7)	11.67 (7)	
Other	5.83 (7)	3.33 (2)	8.33 (5)	
Housing type	
House	47.50 (57)	55.00 (33)	40.00 (24)	0.100
Apartment	52.50 (63)	45.00 (27)	60.00 (36)	
Housing tenure	
Owner-occupied	66.67 (80)	70.00 (42)	63.33 (38)	0.439
Rented	33.33 (40)	30.00 (18)	36.67 (22)	
Indoor plants	
None	10.00 (12)	10.00 (6)	10.00 (6)	0.365
1–9 plants	59.17 (71)	65.00 (39)	53.33 (32)	
≥10 plants	30.83 (37)	25.00 (15)	36.67 (22)	
Cleaning frequency	
1 time/week	42.50 (51)	35.00 (21)	50.00 (30)	0.151
2/3 times/week	30.83 (37)	38.33 (23)	23.33 (14)	
≥ 4 times/week	26.67 (32)	26.67 (16)	26.67 (16)	
Proximity to green areas	
In front of dwelling	38.33 (46)	41.67 (25)	35.00 (21)	0.637
One block away	20.00 (24)	18.33 (11)	21.67 (13)	
≥ Two blocks away	40.83 (49)	38.33 (23)	43.33 (26)	
Floor area (m^2^), median (IQR)	85 (69, 120)	87.5 (60, 120)	85 (75, 115.5)	0.9593
Number of household residents, median (IQR)	3 (2, 3.5)	3 (2, 4)	3 (2, 3)	0.8064
Hours with windows open, mean ± SD	15.17 ±6.11	15.21 ±6.61	15.13 ±5.61	0.9408
Window condensation	
Never	80.00 (96)	75.00 (45)	85.00 (51)	0.513
Almost never	10.83 (13)	13.33 (8)	8.33 (5)	
Sometimes	8.33 (10)	10.00 (6)	6.67 (4)	
Always	0.83 (1)	1.67 (1)	0	
Air conditioning	4.17 (5)	3.33 (2)	5.00 (3)	1.000
Temperature. mean ± SD	26.92 ± 1.58	27.17 ± 1.63	26.66 ± 1.50	0.0795
Relative humidity. median (IQR)	70 (67, 73)	70.5 (67, 73)	71 (67, 73)	0.8660
CO_2_ (ppm). median (IQR)	557 (521; 633)	553 (502.5; 633)	564 (525; 633)	0.3958
Fungal density (CFU/m^3^)	643 (403; 965)	703 (505; 1071)	560 (353; 837)	0.0160
Total DMAT	4.73 ± 8.24	3.57 ± 5.06	5.89 ± 10.42	0.1266
Mold odor	0.5 ± 1.13	0.32 ± 0.81	0.67 ± 1.35	0.0873
Damage and stains	2.44 ± 3.51	2.06 ± 2.64	2.81 ± 4.19	0.2437
Wet or damp conditions	0.79 ± 2.17	0.36 ± 0.78	1.21 ± 2.92	0.0315
Visible mold	0.93 ± 2.28	1.1 ± 2.64	0.76 ± 1.87	0.4274

DMAT, Dampness and Mold Assessment Tool; IQR, interquartile range. P-values were obtained using Pearson's chi-square test, Fisher's exact test, Mann–Whitney U-test, or Student's t-test, as appropriate.

Regarding environmental conditions, the ambient temperature had a median of 26.92 ± 1.58, the relative humidity had a median of 70 (IQR: 67; 73), and the CO_2_ (ppm) had a median of 557 (IQR: 521; 633). When comparing cases and controls, the case participants had an average ambient temperature of 26.66 ± 1.50, while the controls had an average of 27.17 ± 1.63 (*p* = 0.0795).

### 3.4 Environmental molds

**Figure 3:**
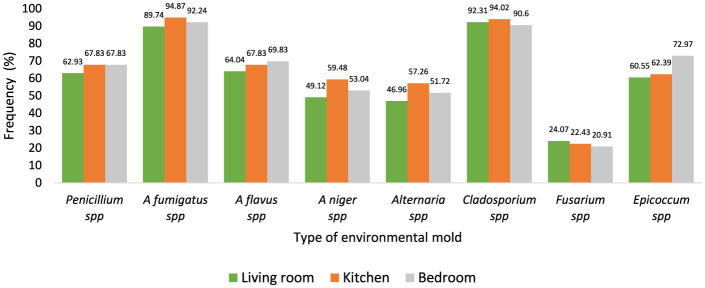
Frequency of identified environmental molds.

*Cladosporium spp*. and *Aspergillus fumigatus* were the most frequently identified environmental molds. No significant differences were observed in mold frequency across sampling areas (living room, kitchen, and bedroom) (see Figure 3). When comparing cases and controls, significant differences were observed in kitchen samples for *Penicillium* spp. and *Fusarium* spp. *Penicillium* spp. was more common among controls (76.27% [45]) than cases (58.93% [33]) (*p* = 0.047), whereas *Fusarium* spp. showed the highest proportion among cases (31.48% [17]) than controls (13.21% [7]) (*p* = 0.023). In bedroom samples, *Penicillium* spp. was also more frequent among controls (79.31% [46]) than cases (56.14% [32]) (*p* = 0.008) (see Supplementary Table 3).

### 3.5 IgE levels

No significant differences were observed between cases and controls in specific IgE levels for *Alternaria alternata, Aspergillus fumigatus*, and *Penicillium chrysogenum* (all *p* > 0.05). For specific IgE levels > 0.10, sensitization to *Alternaria alternata* was observed in 18.64% (11) of cases and 12.07% (7) of controls (*p* = 0.324). Sensitization to *Aspergillus fumigatus* was observed in 18.64% (11) of cases and 8.62% (5) of controls (*p* = 0.115), while sensitization to *Penicillium chrysogenum* was observed in 13.56% (8) of cases and 6.90% (4) of controls (*p* = 0.362). Table 3 also presents the frequency and median levels of the three mold-specific IgE markers.

**Table 3:** Frequency of specific IgE levels for molds among participants.

Specific IgE levels	Total (117)	Controls (58)	Cases (59)	*p*-value
*Alternaria alternata* %(n)	
< 0, 1	84.62 (99)	87.93 (51)	81.36 (48)	0,324
0, 10–0, 25	15, 38 (18)	12, 07 (7)	18, 64 (11)	
Median (IQR)	0, 13 (0, 11, 0, 15)	0, 13 (0, 11, 0, 15)	0, 13 (0, 11, 0, 16)	0, 8462
*Aspergillus fumigatus* %(n)	
< 0, 1	86, 32 (101)	91, 38 (53)	81, 36 (48)	0, 115
0, 10–0, 55	13, 68 (16)	8, 62 (5)	18, 64 (11)	
Median (IQR)	0, 13 (0, 125; 0, 175)	0, 14 (0, 13; 0, 22)	0, 13 (0, 12; 0, 17)	0.1685
*Penicillium chrysogenum*. %(n)	
< 0, 1	89, 74 (105)	93, 10 (54)	86, 44 (51)	0, 362
0, 10–0, 55	10, 26 (12)	6, 90 (4)	13, 56 (8)	
Median (IQR)	0, 125 (0, 12; 0, 15)	0, 13 (0, 12; 0, 145)	0, 125 (0, 115; 0, 185)	0, 9737

IQR, interquartile range. p-values were obtained using Pearson's chi-square test, Fisher's exact test, Mann–Whitney U-test.

### 3.6 Multivariable conditional logistic regression models

The detection of environmental mold (*Fusarium* spp.) in kitchen samples was associated with increased odds of belonging to the case group, although the association was not statistically significant (OR = 3.86; 95% CI: 0.85–17.38). Self-reported nose irritation by SBS was significantly associated with aMCI (OR = 5.84; 95% CI: 1.20–28.37). A history of diabetes also showed a positive but non-significant association with aMCI (OR = 7.68; 95% CI: 0.95–61.16). Conversely, higher environmental temperature (OR = 0.66; 95% CI: 0.42–1.04) and increased frequency of household cleaning exhibited a protective trend against aMCI; however, these associations did not reach statistical significance (Table 4).

**Table 4:** Multivariable conditional logistic regression model for amnestic mild cognitive impairment.

Variable	OR (95% CI)	*p*-value
*Fusarium spp*. in Kitchen Samples	3.86 (0.85; 17.38)	0.078
SBS Nose irritation	5.84 (1.20; 28.37)	0.029
Diabetes Mellitus	7.68 (0.95; 61.16)	0.054
Temperature (1 °C)	0.66 (0.42; 1.04)	0.075
DMAT	0.99 (0.91; 1.08)	0.877
Cleaning frequency	
1 time/week	1	–
2/3 times/week	0.64 (0.19; 2.13)	0.473
≥4 times/week	0.49 (0.14; 1.72)	0.269

Adjusted by design for: age, sex, and socioeconomic stratum. Adjusted in the multivariable model by family history of dementia disease. DMAT, Dampness and Mold Assessment Tool -General Buildings; SBS (Sick Building Syndrome). Pseudo R_2_ = 0.2832, AIC = 66.68, BIC = 87.68.

The models stratified by groups of variables are presented in Supplementary Table 4. Within the group of SBS related characteristics, nose irritation (OR = 3.12; 95% CI: 0.97–10.04) and difficulty concentrating (OR = 1.49; 95% CI: 1.06–2.09) were identified as relevant factors. In the model including specific IgE, no variables reached statistical significance; however, *Aspergillus fumigatus* (IgE ≥ 0.10) showed a positive but non-significant association (OR = 2.34; 95% CI: 0.56–9.78). Regarding mold frequency, *Fusarium* spp. was significantly associated with case status when identified in kitchen samples (OR = 3.09; 95% CI: 0.98–9.70). In the DMAT-based model, wet or damp conditions emerged as a relevant factor. Among environmental measures, higher temperature showed a protective effect (OR = 0.68; 95% CI: 0.50–0.93). Finally, in the model including only housing characteristics, apartment-type housing and higher frequency of household cleaning was inversely associated with aMCI, although the associations was not statistically significant, Relative to participants who performed general household cleaning once per week, those cleaning 2–3 times per week exhibited lower odds of aMCI (OR = 0.43; 95% CI: 0.17–1.07), while participants cleaning ≥4 times per week also demonstrated reduced odds (OR = 0.83; 95% CI: 0.32–2.15).

## 4 Discussion

The results of this study suggest that intradomiciliary biological factors and housing characteristics may be associated with aMCI in older adults. This is the first (or one of the first) studies to analyze these relationships in an older adult population in an urban context in a Latin American city, using a combination of physical and biological measurements with questionnaires to comprehensively approach the study of the external and internal exposome related to aMCI.

Among the identified molds, the presence of *Fusarium* spp. in kitchen samples showed a positive, but non-significant association with aMCI. This genus has previously been linked to building-related illnesses. Subsequent research has highlighted the role of mycotoxins in neuropsychiatric symptoms and immune system dysregulation, particularly for molds such as *Trichoderma, Fusarium*, and *Stachybotrys* (21). More recent studies have further explored the impact of mycotoxin exposure, reporting associations between toxins produced by *Fusarium spp*. including zearalenone, fumonisin B1, deoxynivalenol, T-2 toxin, and patulin, and the development of neurodegenerative and psychological disorders (52). In a study conducted in Spain, mycotoxins were analyzed in blood samples from individuals with dementia and healthy controls, identifying differences in ochratoxin A and sterigmatocystin levels. However, the authors noted important limitations, including a small sample size and age disparities between groups (53). Nevertheless, it is important to highlight that the biological impact may be related to the production of mycotoxins and pro-inflammatory compounds capable of inducing oxidative stress and neuroinflammation mechanisms widely recognized in the pathophysiology of cognitive impairment. (54–56).

Regarding the perception of symptoms related to sick building syndrome (SBS), nose irritation was associated with aMCI. It is important to note that air pollution has been reported to exert adverse health effects, including the induction of inflammatory processes and cytokine production. These mediators may cross the blood–brain barrier and contribute to neurotoxicity (57). Regarding the frequency of SBS, specifically nose irritation, the frequency observed in our study was higher than that reported in a study conducted in China, which found prevalence of 3.1% in 2010 and 2.3% in 2019 (58). However, it was comparable to more recent estimates reported in China in 2022 (16.5%) (39) and in the Karaj River region, Iran (14.9%) (41). A plausible biological mechanism underlying these associations involves the entry of microorganisms through the nasal cavity, with subsequent ascent along the olfactory pathway via the olfactory bulb, crossing the cribriform plate to reach the central nervous system. This route has been proposed as a pathway for viruses, bacteria, and toxins (59), where they may trigger inflammatory responses and contribute to neuronal damage (60).

A history of DM among participants showed higher estimated odds of aMCI, however, the association was not statistically significant, and the estimate was imprecise, as reflected by the wide confidence interval. Nevertheless, this finding is consistent with evidence from a systematic review and meta-analysis reporting a high global prevalence of type 2 DM among individuals with MCI, with a higher frequency observed in women (61, 62). Notably, individuals with type 2 DM exhibit alterations in immune response, including impaired pathogen control, which may increase susceptibility to infections (63). Therefore, the observed association should be interpreted with caution and warrants confirmation in larger studies.

Temperature and relative humidity have been associated with a significant increase in indoor mold spore concentrations (64, 65). In the present study, higher-than-recommended indoor environmental parameters were observed, particularly with respect to relative humidity, for which recommended levels typically range between 40 and 60% (66, 67).

Regarding housing-related structural conditions, the frequency of damage and stains, visible mold, and wet or damp conditions was slightly higher than that reported by Avendaño (68) in a study conducted in Ocaña, Norte de Santander. In that study, 43% of dwellings presented moisture-related damage, 9% visible mold, 45% black stains, and 16% paint deterioration. Notably, within the semi-quantitative DMAT index, the wet or damp conditions indicator was significantly associated with aMCI in the stratified models. Moreover, recent evidence suggests that relative humidity may play a more critical role than temperature in promoting the growth of indoor molds (69).

In the present study, cases tended to report a higher frequency of perceived mold odor compared with controls; however, this difference did not reach statistical significance in the bivariate analysis (10.91% in bedrooms of cases vs. 3.7% in controls; *p* = 0.140) and was not retained in the multivariable model. Nevertheless, findings from a cohort study conducted in China by Liu (70) indicate that the perception of indoor dampness odor is associated with MCI (70).

The frequency of household cleaning was identified as a protective factor for aMCI. Increasing attention has been directed toward indoor air quality, particularly regarding the potential adverse effects of indoor air pollution on neurological health and cognitive function. Key contributing factors include the presence of fungi and their metabolites, particulate matter, housing structural conditions, and residents' behaviors (71–73). However, further evidence is still needed to clarify and confirm these associations (74).

In the stratified multivariable analysis, specific IgE levels ≥0.10 to *Aspergillus fumigatus and Alternaria alternata* showed estimated probabilities of belonging to the aMCI case group, but no statistically significant association was observed. To our knowledge, no studies have directly evaluated the association between these IgE levels and cognitive outcomes. However, a study conducted in Turkey among children with a history of allergic rhinitis assessed clinical symptoms in relation to pollen exposure, mold sensitization, and IgE measurements; nasal irritation was among the most frequently reported symptoms (75). Additionally, a case–control study in Belgium (76) evaluated total IgE and specific IgE to *Aspergillus fumigatus, Alternaria alternata*, and *Penicillium* spp., correlating these with colony-forming unit (CFU) counts, and identified mold contamination in 90% of dwellings among patients with asthma.

Among the strengths of this study, participants with aMCI underwent a comprehensive clinical evaluation aimed at excluding psychological conditions such as depression and anxiety, which may act as potential confounders in the identification of this diagnosis (77). Another strength relates to the air sampling procedure: the use of equipment that collects a standardized volume of air allows for more reliable comparisons across samples. Additionally, the culture medium employed favored the growth of environmental molds. However, some limitations should be acknowledged. More advanced techniques, such as molecular identification methods, could provide greater precision in the characterization of mold species (78).

However, some limitations should be mentioned. First, the sample size was calculated based on previous reports of exposed and unexposed participants and the development of conditions such as brain fog, headache, and mean and standard deviation scores on neurocognitive tests (symbol and digit span tests, memory, vocabulary). Subsequently, when the analyses were performed, measures of association with wide confidence intervals were identified in the multivariate model (diabetes mellitus, SBS nose irritation, and Fusarium spp.), where prevalences in the control group ranged from 10 to 20%. We recognize that a larger sample size provides greater statistical power and more precise effect estimates. The relatively modest sample size of the present study limits the evaluation of some variables in the multivariate analyses. We encourage future studies with larger populations to confirm and expand upon these findings.

Second, the study exhibits potential selection bias in the control group due to non-random selection; controls were identified partly through case referral/convenience sampling, which could produce controls with similar environments or social characteristics (79, 80). We conducted an additional multivariable analysis adjusting for educational level, socioeconomic status, housing type, municipality of residence, measures of relative humidity, and CO_2_. The effect estimates for Fusarium spp. in kitchen samples, indoor temperature, DMAT and cleaning frequency remained similar in both magnitude and direction after adjustment, suggesting that these associations were relatively robust to differences in measured socioeconomic and residential characteristics. For SBS nose irritation and diabetes mellitus, the adjusted odds ratios increased, and the corresponding 95% confidence intervals became wider, indicating greater uncertainty in the estimates, likely related to the limited sample size and sparse data in some categories (Supplementary Table 5). Furthermore, we acknowledge that individuals living in households classified as low socioeconomic strata were underrepresented in the study sample, which may have affected the representativeness of the findings.

Third, dietary intake was not assessed and may represent a source of residual confounding. This limitation is relevant because certain foods, including spices, herbs, and nuts, have been identified as potential sources of mold contamination and mycotoxin exposure (81). At the same time, several dietary patterns and nutritional factors, including the Mediterranean diet, DASH diet, MIND diet, B vitamins, antioxidants, omega-3 fatty acids, nuts, fruits, and vegetables, have been associated with a lower risk of cognitive decline and aMCI (82). Consequently, dietary habits could potentially influence both environmental exposure profiles and cognitive outcomes.

Occupational exposure was not specifically assessed in the present study. Nevertheless, more than 70% of participants were women, and the most frequently reported current occupation was homemaking. Participants engaged in self-employment or other occupations accounted for less than 35% of the study population. Although previous studies have linked occupational mold exposure with neurological symptoms (24), evidence specifically relating occupational exposure to aMCI remains limited. Future studies should incorporate a more detailed assessment of occupational history and workplace exposures. This consideration is particularly relevant given recent evidence suggesting associations between environmental exposures, including solid fuel use, and cognitive impairment (83).

Additional factors associated with cognitive impairment, such as APOE 3/4 genotype (84), sleep quality (85), social engagement (86), physical activity, and other lifestyle characteristics, were not evaluated. However, recent meta-analytic evidence indicates that advancing age, lower educational attainment, and depression are among the strongest risk factors for aMCI (87). Educational level was assessed and considered in the analyses, and all participants underwent a psychiatric evaluation conducted by a physician specialized in psychiatry to identify active major depressive disorders. Nevertheless, residual confounding from unmeasured or incompletely measured factors remains possible. Therefore, the observed associations should be interpreted with appropriate caution and confirmed in future studies incorporating a broader assessment of behavioral, genetic, occupational, and lifestyle determinants.

In addition, the study may be subject to information bias, as participants could have felt judged or reluctant to disclose or allow full inspection of areas affected by mold within their homes, despite being informed of the importance of evaluating all relevant spaces, including the living room, kitchen, and bedroom.

Finally, although an effort was made to recruit cases from a population-based setting, the sampling strategy did not correspond to simple random sampling but rather to convenience sampling, as some participants suggested potential controls or individuals from their close social environment were contacted (88).

For future research, the incorporation of mycotoxin analyses, ideally using blood samples, would be highly relevant, as these may better reflect chronic exposure, in contrast to urine samples, which are more indicative of recent or acute exposure (54, 55, 89). The identification of mycotoxins in built environments using mass spectrometry has been addressed in recent reviews, which highlight that one of the main challenges lies in the development of robust and reliable analytical methods. Ensuring quality across all stages, sampling, sample preparation, and analysis, is critical and requires highly trained personnel and specialized resources (90). Available evidence is largely derived from high-income countries, including France, Italy, Croatia, Poland, Denmark, Germany, Finland, and the United States, where techniques such as liquid chromatography–mass spectrometry (LC–MS), gas chromatography mass spectrometry (GC–MS), and solid-phase extraction coupled with mass spectrometry have been employed. Future studies should prioritize the development of rapid, sensitive, and multiplex analytical tools capable of simultaneously detecting a broad spectrum of mycotoxins within a single matrix, with lower limits of detection and greater applicability across diverse environmental settings.

While fungal identification in this study was based on microscopic examination, future studies should consider incorporating molecular techniques such as qPCR and DNA metabarcoding (91). These methods could provide a more detailed characterization of indoor fungal communities. Additionally should consider incorporating biodiversity indicators derived from metagenomic approaches (92). An illustrative example is a case–control study conducted in Singapore (93), in which indoor air samples were collected from bedrooms and balconies using SASS3100 (Research International) filter-based air samplers operating at a flow rate of 100 L·min^−1^ for eight consecutive hours. Sequencing was performed using the Illumina HiSeq 2,500 platform (Illumina). Similar approaches have been applied in other settings, including studies conducted in Qatar (94) and in a burn intensive care unit (95), highlighting the feasibility and relevance of metagenomic techniques for characterizing microbial diversity in built environments.

Future studies should also consider focusing on specific mold species, such as *Stachybotrys* spp. (96), commonly referred to as “black mold,” which has been associated with sick building syndrome and respiratory conditions. This genus is recognized for producing a wide range of mycotoxins, including satratoxins (F, G, and H), isosatratoxin F, roridins (A, E, H, and L-2), and verrucarins (A and J) (97, 98). These compounds have been linked to neurotoxic effects, further supporting their potential relevance in studies of cognitive impairment (99).

Furthermore, the development of strategies to raise awareness about moisture control is essential. Key measures include preventing condensation, improving ventilation, and maintaining adequate indoor temperature, all of which are critical for controlling dampness and mold growth. Practical actions include the use of exhaust fans, opening windows in kitchens and bathrooms, thoroughly drying wet surfaces, and repairing sources of water leakage. Hard surfaces should be cleaned with water and detergent and dried completely, and materials heavily affected by mold, such as ceiling panels, carpets, and furniture, should be replaced when necessary. Individuals experiencing symptoms potentially related to mold exposure should seek medical evaluation. In cases of extensive water damage or mold contamination, the use of appropriate personal protective equipment is recommended.

Further research is warranted to clarify the causal nature of this relationship. Establishing causality would provide a stronger scientific basis for implementing targeted interventions aimed at reducing exposure and promoting healthier indoor environments for older adults. Such measures include remediation of areas affected by mold and dampness, improvements in ventilation, lighting, and household cleaning practices, as well as routine monitoring of indoor air quality. Prompt repair of structural deficiencies contributing to moisture accumulation is also essential (78).

## 5 Conclusion

In the study population, factors associated with aMCI included the perception of nasal irritation while inside the home that improved upon leaving, it was the only factor significantly, the presence of environmental mold (*Fusarium* spp.), and a history of diabetes mellitus (DM) also associated with higher estimated odds of aMCI, but not statistically significant and imprecise. In contrast, higher indoor temperature and greater frequency of household cleaning were associated with lower odds of aMCI, however, these associations did not reach statistical significance. These findings highlight the need for further research to elucidate the role of residential environmental exposures with aMCI.

From a mechanistic perspective, chronic exposure to indoor pollutants, including molds and their metabolites, may contribute to inflammatory responses and neurotoxic pathways, while underlying metabolic conditions such as DM may further increase vulnerability. Conversely, environmental conditions that reduce dampness and microbial growth, such as adequate temperature control and regular cleaning, may help mitigate these risks.

Overall, these results underscore the importance of considering residential environmental factors in the context of cognitive health and highlight the need for further longitudinal and mechanistic studies to clarify causal relationships and inform targeted preventive strategies aimed at promoting healthy aging.

## Data Availability

The original contributions presented in the study are included in the article/Supplementary material, further inquiries can be directed to the corresponding authors.
