# Supplementary material for: Association between environmental mold exposure and mild cognitive impairment in older adults: a case–control study
Source: Front Public Health. 2026 Jul 13;14:1877177. doi: 10.3389/fpubh.2026.1877177 (PMC13402551; doi:10.3389/fpubh.2026.1877177)
Supplement: Supplementary file 1 [file Table_1.docx]

**Supplementary tables**

Suplementary table 1. Characteristics of Instruments Used to Measure Cognitive Impairment

| Test | Instrument | Time | Items | Components |
| --- | --- | --- | --- | --- |
| **MMSE**  Mini-Mental State Examination(1) | Interview  Paper-and-pencil | 10 minutes | 0-30/11  Cutoff score=24 | 5 subscales: orientation, attention/concentration, memory, language, and visuospatial ability |
| **MoCA**  Montreal Cognitive Assessment(1) | Interview  Paper-and-pencil | 10 minutes | 0-30/11  Cutoff score =21 | 6 subscales: orientation, memory, language, attention, concentration, and functional performance. |
| Neuropsychological Test | | | | |
| Hoopkins verbal learnign test(2) | Paper-and-pencil Stopwatch | 3 trials for recall and one for delayed recall (20–25 minutes) | Report  # correct responses (#/12)  # semantically related false positives (#/6)  # semantically unrelated false positives (#/6)  # total false positives (errors) (#/12)  Cutoff scores were defined as= 3 for delayed recall and 13 for immediate recall. | The test evaluates total recall, delayed recall, retention percentage, and recognition discrimination index. |
| Digit Symbol Test (3) | Paper-and-pencil | 5 minutes | Written score  Oral score  In individuals aged 56–76 years, the mean score was 27 ± 12.1. | The test evaluates visual perception, stimulus recognition, attention (focused, selective, and sustained), task monitoring, and interference control. |
| Trail Making Test Part A and Part B (4) | Paper-and-pencil Stopwatch | Part A was discontinued after 100 seconds and Part B after 300 seconds. Prior to testing, participants completed a training session. | Time required to complete the test.  Among individuals aged 56–75 years, the mean completion time was:  Form A: 75.8 ± 22.8 seconds  Form B: 178.8 ± 80.7 seconds | Working memory |
| Verbal Fluency Test (5) | Paper-and-pencil | Participants were asked to name as many animals as possible within one minute and to generate words beginning with the letters F, A, S, or M. | Number of words generated.  Mean scores were as follows:  Letter “F”: 9.9 ± 4.3  Letter “A”: 10.1 ± 4.4  Letter “S”: 9.6 ± 4.4  “Animals”: 15.6 ± 4.5  “Fruits”: 13.0 ± 3.7 | Semantic verbal fluency (animals)  Phonemic verbal fluency (F, A, S, M)  Clustering: semantic memory networks (temporal cortex activity)  Switching: efficient search and cognitive flexibility (prefrontal cortex activity) |
| Stroop Color and Word Test (6)(7) | Paper-and-pencil Stopwatch | The task consisted of three conditions: Word (W), Color (C), and Word–Color (W–C), each administered for 45 seconds. | The number of correctly generated responses was recorded following a training phase. Scores were adjusted for participants’ age. Mean scores were as follows: Word = 77.78 ± 21.8;  Color = 55.58 ± 16.6;  Word–Color = 30.88 ± 11.6; Interference = −1.48 ± 9.1. | Executive functions: mental flexibility, selective attention, processing speed, and response inhibition |
| Rey–Osterrieth Complex Figure Test (8) | Paper-and-pencil Stopwatch | 60 minutes | >7  The figure is composed of 18 elements.  In individuals aged 56–76 years,  *Copying task*= 27.98±7.4  *Log-term memory*= 13.78±7.4 | Visuospatial memory  Executive functions  Psychomotor function  Immediate visual memory  Attention |
| Token Test (9) | Paper-and-pencil Images of circles and squares in different colors:  R: Red; N: Black; Y: Yellow; W: White; G: Green |  | Scores are interpreted according to age-specific ranges:  50–56 years: 31.5–32.0  57–59 years: 32.0–32.5  60–68 years: 32.0–32.5  69–71 years: 31.0–31.5  72–74 years: 31.5–32.5 | Language  Working memory |

Note: Instruments used to assess amnestic mild cognitive impairment (aMCI). Adapted from: Scott J y Mayo A., 2018 (1)

**Supplementary table 2. Structural Housing Conditions Comparing Cases and Controls**

| Variable | Total | Controls | Cases | *p* value |
| --- | --- | --- | --- | --- |
|  | (120) | (60) | (60) |  |
| Window condition |  |  |  | 1.000 |
| Poor | 0.83(1) | 1.67(1) | 0 |  |
| Fair | 6.67(8) | 6.67(4) | 6.67(4) |  |
| Good | 44.17(53) | 43.33(26) | 45.00(27) |  |
| Excellent | 48.33(58) | 48.33(29) | 48.33(29) |  |
| Roof condition |  |  |  | 0.407 |
| Poor | 2.50(3) | 0 | 5.00(3) |  |
| Fair | 9.17(11) | 8.33(5) | 10.00(6) |  |
| Good | 24.17(29) | 26.67(16) | 21.67(13) |  |
| Excellent | 64.17(77) | 65.00(39) | 63.33(38) |  |
| Wall condition |  |  |  | 0.771 |
| Poor | 1.67(2) | 1.67(1) | 1.67(1) |  |
| Fair | 5.83(7) | 3.33(2) | 8.33(5) |  |
| Good | 25.0(30) | 26.67(16) | 23.33(14) |  |
| Excellent | 67.50(81) | 68.33(41) | 66.67(40) |  |
| Water supply condition |  |  |  | 1.000 |
| Poor | 0 | 0 | 0 |  |
| Fair | 3.33(4) | 3.33(2) | 3.33(2) |  |
| Good | 20.83(25) | 21.67(13) | 20.00(12) |  |
| Excellent | 75.83(91) | 75.00(45) | 76.67(46) |  |
| Sewage system condition |  |  |  | 0.711 |
| Poor | 0.83(1) | 0 | 1.67(1) |  |
| Fair | 2.50(3) | 1.67(1) | 3.33(2) |  |
| Good | 15.83(19) | 18.33(11) | 13.33(8) |  |
| Excellent | 80.83(97) | 80.00(48) | 81.67(49) |  |

**Supplementary table 3. Prevalence of Identified Environmental Molds by Area**

| Variable | Total | Controls | Cases | *p-*value |
| --- | --- | --- | --- | --- |
|  | (120) | (60) | (60) |  |
| Living room |  |  |  |  |
| *Penicillium* spp | 62.93 (73) | 71.19 (42) | 54.39 (31) | 0.061 |
| *A fumigatus* spp | 89.74 (105) | 91.53 (54) | 87.93 (51) | 0.522 |
| *A flavus* spp | 64.04 (73) | 70.18 (40) | 57.89 (33) | 0.172 |
| *A niger* spp | 49.12 (56) | 50.88 (29) | 47.37 (27) | 0.708 |
| *Alternaria* spp | 46.96 (54) | 50 (29) | 43.86 (25) | 0.509 |
| *Cladosporium* spp | 92.31 (108) | 94.92 (56) | 89.66 (52) | 0.286 |
| *Fusarium* spp | 24.07 (26) | 18.52 (10) | 29.63 (16) | 0.177 |
| *Epicoccum* spp | 60.55 (66) | 68.52 (37) | 52.73 (29) | 0.092 |
| Kitchen |  |  |  |  |
| *Penicillium* spp | 67.83 (78) | 76.27 (45) | 58.93 (33) | 0.047 |
| *A fumigatus* spp | 94.87 (111) | 96.61 (57) | 93.1 (54) | 0.390 |
| *A flavus* spp | 67.83 (78) | 72.41(42) | 63.16 (36) | 0.323 |
| *A niger* spp | 59.48 (69) | 67.24 (39) | 51.72 (30) | 0.089 |
| *Alternaria* spp | 57.26 (67) | 54.24 (32) | 60.34 (35) | 0.504 |
| *Cladosporium* spp | 94.02 (110) | 96.61 (57) | 91.38 (53) | 0.233 |
| *Fusarium* spp | 22.43 (24) | 13.21 (7) | 31.48 (17) | 0.023 |
| *Epicoccum* spp | 62.39 (68) | 70.91 (39) | 53.7 (29) | 0.064 |
| Bedroom |  |  |  |  |
| *Penicillium* spp | 67.83 (78) | 79.31 (46) | 56.14 (32) | 0.008 |
| *A fumigatus* spp | 92.24 (107) | 94.92 (56) | 89.47 (51) | 0.273 |
| *A flavus* spp | 69.83 (81) | 70.69 (41) | 68.97 (40) | 0.840 |
| *A niger* spp | 53.04 (61) | 62.07 (36) | 43.86 (25) | 0.050 |
| *Alternaria* spp | 51.72 (60) | 54.24 (32) | 49.12 (28) | 0.582 |
| *Cladosporium* spp | 90.6 (106) | 94.92 (56) | 86.21 (50) | 0.107 |
| *Fusarium* spp | 20.91 (23) | 18.87 (10) | 22.81 (13) | 0.612 |
| *Epicoccum* spp | 72.97 (81) | 78.18 (43) | 67.86 (38) | 0.221 |

P-values were obtained using Pearson’s chi-square test

**Supplementary table 4.** Multivariable Conditional Logistic Regression Models for aMCI, Stratified by Variable Groups

| Variable | OR (IC95%) | p-value | Pseudo R2 | AIC | BIC |
| --- | --- | --- | --- | --- | --- |
| **General characteristics** |  |  | 0.1509 | 79.91 | 98.94 |
| Occupation |  |  |  |  |  |
| Housekeeper | 1 |  |  |  |  |
| Employed | 0.13(0.01; 1.53) | 0.106 |  |  |  |
| Self-employed | 0.5(0.08; 1.55) | 0.170 |  |  |  |
| Retired | 1.36(0.46; 4.03) | 0.570 |  |  |  |
| Diabetes Mellitus | 2.06(0.542; 8.16) | 0.300 |  |  |  |
| Hypertension | 0.76(0.29; 1.95) | 0.572 |  |  |  |
| Family history of Alzheimer’s disease | 1.52(0.57; 4.06) | 0.394 |  |  |  |
| Smoking | 1.29(0.44; 3.78) | 0.638 |  |  |  |
| **SBS** |  |  | 0.1563 | 74.17 | 79.75 |
| Nose irritation | 3.12(0.97; 10.04) | **0.056** |  |  |  |
| Difficulty concentrating | 1.49(1.06; 2.09) | **0.019** |  |  |  |
| **Specific IgE Levels ≥ 0.10** |  |  | 0.0489 | 81.15 | 89.35 |
| *Alternaria alternanta* | 1.63(0.45; 5.91) | 0.453 |  |  |  |
| *Aspergillus fumigatus* | 2.34(0.56; 9.78) | 0.243 |  |  |  |
| *Penicillium Chrysogenum* | 1.25(0.26; 5.94) | 0.772 |  |  |  |
| **Prevalence mold** |  |  | 0.1565 | 65.63 | 73.50 |
| *Penicillium spp*. in Kitchen Samples | 0.52(0.14; 1.97) | 0.346 |  |  |  |
| *Fusarium spp.* in Kitchen Samples | 3.09(0.98; 9.70) | **0.056** |  |  |  |
| *Penicilliuim spp.* in Bedroom Samples | 0.54(0.17; 1.69) | 0.291 |  |  |  |
| **DMAT** |  |  | 0.1268 | 78.21 | 89.22 |
| Mold odor | 1.29(0.69; 2.43) | 0.416 |  |  |  |
| Damage and stains | 1.04(0.84; 1.29) | 0.685 |  |  |  |
| Wet or damp conditions | 1.76(1.02; 3.06) | **0.042** |  |  |  |
| Visible mold | -0.65(0.39; 1.09) | 0.110 |  |  |  |
| **Environmental Measures** |  |  | 0.0911 | 81.59 | 89.95 |
| CO₂ (1ppm). | 1.00(0.99; 1.00) | 0.617 |  |  |  |
| Temperature (1°C) | 0.68(0.50; 0.93) | **0.018** |  |  |  |
| Relative humidity (1%) | 0.89(0.78; 1.01) | 0.077 |  |  |  |
| **House Characteristic** |  |  | 0.0797 | 82.54 | 90.91 |
| Housing type |  |  |  |  |  |
| House | 1 |  |  |  |  |
| Apartment | 2.00(0.87; 4.60) | 0.099 |  |  |  |
| Cleaning frequency |  |  |  |  |  |
| 1 time/week | 1 |  |  |  |  |
| 2/3 times/week | 0.43(0.17; 1.07) | 0.071 |  |  |  |
| ≥ 4 times/week | 0.83(0.32; 2.15) | 0.716 |  |  |  |

All models were adjusted for age, sex, and socioeconomic stratum by design. AIC: Akaike Information Criterion. BIC: Bayesian Information Criterion. DMAT (Dampness and Mold Assessment Tool -General Buildings)

**Supplementary table 5.** Multivariable Conditional Logistic Regression Models for aMCI – sensitivity analysis

| Variable | OR (IC95%) | p-value |
| --- | --- | --- |
| *Fusarium spp.* in Kitchen Samples | 6.87 (0.63; 74.89) | 0.114 |
| SBS Nose irritation | 67.36 (3.14; 1142.95) | 0.007 |
| Diabetes Mellitus | 13.28(0.55; 317.47) | 0.110 |
| Temperature (1°C) | 0.50(0.25; 1.00) | 0.050 |
| DMAT | 1.10(0.88; 1.38) | 0.397 |
| Cleaning frequency |  |  |
| 1 time/week | 1 |  |
| 2/3 times/week | 0.30(0.04; 1.87) | 0.201 |
| ≥ 4 times/week | 0.19(0.02; 1.61) | 0.129 |

Adjusted by design for: age, sex, and socioeconomic stratum. Adjusted in the multivariable model by family history of dementia disease, educational level, socioeconomic status, housing type, municipality of residence, measures of relative humidity and CO2. DMAT (Dampness and Mold Assessment Tool -General Buildings). Pseudo R2=0.2832, AIC=68.40, BIC=113.02

**References supplementary table 1**

1. Scott J, Mayo AM. Instruments for detection and screening of cognitive impairment for older adults in primary care settings: A review. Geriatr Nurs (Minneap) [Internet]. 2018;39(3):323–9. Available from: https://doi.org/10.1016/j.gerinurse.2017.11.001

2. Cherner M, Suarez P, Lazzaretto D, Artiola L, Rivera M, Dawes S, et al. Demographically corrected norms for the Brief Visuospatial Memory Test-revised and Hopkins Verbal Learning Test-revised in monolingual Spanish speakers from the U . S .– Mexico border region. Arch Clin Neuropsycology [Internet]. 2007;22:343–53. Available from: https://doi.org/10.1016/j.acn.2007.01.009

3. Ramos-Galarza C, Acosta-Rodas P, Jadán-Guerrero J, Guevara-Maldonado CB, Zapata-Rodríguez M, Apolo-Buenaño D. Neuropsychological Assessment of Attention : Symbols And Digits Test . Rev Ecuatoriana Neurol [Internet]. 2018;27(1). Available from: http://scielo.senescyt.gob.ec/pdf/rneuro/v27n1/2631-2581-rneuro-27-01-00030.pdf

4. Reitan RM. Validity of the making test as an indicator of organic brain damage. Percept Mot Skills [Internet]. 1958;271–6. Available from: 10.2466/pms.1958.8.3.271

5. Uribe-Pérez A, Flores-Vázquez PDJF, Mondragón-Maya PDA. Ejecución en el Test de Fluidez Verbal en pacientes con Trastorno Neurocognitivo Leve Verbal fluency performance in patients with Mild Neurocognitive Disorder. Rev Ecuatoriana Neurol [Internet]. 2022;31(3):33–40. Available from: http://scielo.senescyt.gob.ec/pdf/rneuro/v31n3/2631-2581-rneuro-31-03-00033.pdf

6. Periáñez JA, Lubrini G, García-gutiérrez A, Ríos-lago M. Construct Validity of the Stroop Color-Word Test : Influence of Speed of Visual Search , Verbal Fluency , Working Memory , Cognitive Flexibility , and Conflict Monitoring. Arch Clin Neuropsychol. 2020;00:1–13.

7. Rodriguez L, Pulido N del C, Pineda C. Propiedades psicométricas del Stroop , test de colores y palabras en población colombiana no patológica *. Univ Psychol. 2016;15(2):255–72.

8. Sala JI, Carboenll X. Análisis del uso de estrategias complejas de personas con discapacidad intelectual en un contexto de visoconstrucción. Rev Española Discapac. 2021;135–52.

9. Steinberg BA, Bieliauskas LA, Smith GE, Langellotti C, Ivnik R. Mayo ’ s Older Americans Normative Studies : Age- and IQ-Adjusted Norms for the Boston Naming Test , the MAE Token Test , and the Judgment of Line Orientation Test. Clin Neuropsychol [Internet]. 2005;19(3–4):280–328. Available from: http://dx.doi.org/10.1080/13854040590945229
